# Pre-exercise isomaltulose intake affects carbohydrate oxidation reduction during endurance exercise and maximal power output in the subsequent Wingate test

**DOI:** 10.1186/s13102-023-00702-7

**Published:** 2023-07-24

**Authors:** Naoko Onuma, Daisuke Shindo, Eriko Matsuo, Miki Sakazaki, Yukie Nagai, Kentaro Yamanaka

**Affiliations:** 1 School of Pharmacy, Nihon University, Funabashi, Chiba Japan; 2 Graduate School of Life Sciences, Showa Women’s University, Tokyo, Japan; 3 College of Sports Sciences, Nihon University, Tokyo, Japan; 4 Mitsui DM Sugar Co. Ltd, Tokyo, Japan

**Keywords:** Isomaltulose, Endurance exercise, Carbohydrate oxidation, Energy expenditure, Anaerobic power output

## Abstract

**Background:**

Ingestion of low-glycemic index (GI) isomaltulose (ISO) not only suppresses subsequent carbohydrate (CHO) oxidation but also inversely retains more CHO after prolonged endurance exercise. Therefore, ISO intake may affect anaerobic power output after prolonged endurance exercise. This study aimed to clarify the time course of CHO utilization during endurance exercise after a single intake of ISO or sucrose (SUC) and the anaerobic power output at the end of endurance exercise.

**Methods:**

After an intake of either ISO or SUC, 13 athletes were kept at rest for 60 min. Thereafter, they performed a 90-min of treadmill running at their individual target level of % ${\rm{\dot V}}{{\rm{O}}_2}$max. During the experimental session, the expired gas was recorded, and the energy expenditure (EE) and CHO oxidation rate were estimated. Immediately after 90 min of running, a 30-s Wingate test was performed, and the maximal anaerobic power output was compared between the ISO and SUC conditions.

**Results:**

The percentage of CHO-derived EE increased rapidly after CHO intake and then decreased gradually throughout the experiment. The slopes of the regression lines calculated from the time course in the CHO-derived EE were significantly (negatively) larger in the SUC condition (-19.4 ± 9.6 [%/h]) than in the ISO condition (-13.3 ± 7.5 [%/h]). Furthermore, the maximal power output in the Wingate test immediately after the endurance exercise was significantly higher in the ISO condition than in the SUC condition (peak power: 12.0 ± 0.6 vs. 11.5 ± 0.9 [W/kg]).

**Conclusion:**

Compared with SUC intake, ISO intake does not produce an abrupt decline in the percentage of CHO-derived EE during prolonged endurance exercise; it remains relatively high until the final exercise phase. Additionally, anaerobic power output at the end of the exercise, largely contributed by anaerobic glycolysis, was greater after ISO intake than after SUC intake.

## Introduction

The main energy sources for human whole-body skeletal muscles during endurance exercise are carbohydrates (CHO) and fat. Depending on the amount of available endogenous and exogenous CHO substrates, the proportion of CHO and fat utilization can be adapted [1–3]. For example, with increasing exercise duration, the utilizable CHO substrate gradually decreases and the proportion of fat utilization increases [4–6]. Several studies have demonstrated that CHO intake before and/or during endurance exercise improves or decreases exercise performance, depending on the type, amount, timing, and frequency of CHO intake [7–12].

Isomaltulose (ISO) is a disaccharide containing glucose and fructose linked by a 1,6-glycosidic bond [13]. Because ISO is completely digestible, but the rate of digestion is slow, the glucose and fructose digested from ISO are absorbed slowly, resulting in a low glycemic index (GI = 32, [14]). Intake of low-GI meals, compared with high-GI meals, suppresses sudden postprandial elevations in blood glucose levels and results in a lower insulin response [15–17]. Several studies have shown that ISO intake before and/or during prolonged exercise in trained individuals suppresses blood glucose spikes and insulin responses, resulting in stable blood glucose levels throughout the exercise [18–23]. These results indicate that CHO oxidation is suppressed and fat oxidation is promoted during the early phase of prolonged exercise. It also suggests the possible beneficial effects of retainment of depleting CHO stores in the final phase of prolonged exercise.

Time-trial cycling performance for tens of minutes after prolonged exercise with ISO intake reportedly have produced inconsistent results: the performance improved [20], remained unchanged [21], or decreased [22]. In addition, ISO intake does not significantly affect soccer-specific movements, which require speed and fine skill, after endurance exercise [23]. Furthermore, to the best of our knowledge, the effects of ISO intake on the maximum anaerobic power output after prolonged exercise have not yet been evaluated. In competitive athletes, the maximum output of anaerobic power in the final phase of prolonged exercise (e.g., just before the finish line in long-distance running or during additional time in the second half of a soccer game) is important; it often makes a difference between winning and losing a competition.

The Wingate anaerobic test is a 30-s all-out cycling test in which the work done is generally used as a measure of anaerobic capacity [24]. The power output during the Wingate test reportedly depends on the total energy released from three systems: adenosine triphosphate-phosphocreatine (ATP-PCr) system, anaerobic glycolysis, and aerobic processes [25–28]. The contribution of the ATP-PCr system to the power output is large in the first few seconds. Thereafter, the contribution of anaerobic glycolysis becomes dominant, and the contribution of aerobic processes gradually increases. Aerobic processes utilize both CHO and fat as substrates, whereas anaerobic glycolysis utilizes only CHO. Thus, the extent that anaerobic glycolysis contributes to the power output during the Wingate test can be significantly affected by the amount of available CHO. This implies that Wingate test performance after prolonged endurance exercise might be affected by low-GI ISO intake, which can maintain relatively high blood glucose levels.

In this study, we aimed to determine the time course of CHO utilization during endurance exercise after ISO or sucrose (SUC) intake and the performance of maximal anaerobic power output at the end of exercise. We hypothesized that the time course of CHO utilization differed between the test beverages; Compared with SUC intake, in ISO intake, the postprandial CHO oxidation increase immediately after intake and continuous decrease during endurance exercise might be suppressed, resulting in a relatively high CHO oxidation at the end of the exercise. If so, the performance in the Wingate anaerobic test immediately after 90 min of endurance exercise might be affected by the test beverage because of the different amounts of CHO available at the end of the exercise.

## Methods

### Participants

Thirteen young male athletes from various sports (age: 21.5 ± 2.1 years, height: 174.4 ± 6.3 cm, weight: 63.5 ± 6.7 kg, BMI: 20.9 ± 1.4 kg/m^2^, maximal oxygen consumption [${\rm{\dot V}}{{\rm{O}}_2}$max]: 62.0 ± 6.4 mL/kg/min) volunteered to participate in this study. They were recruited from a university or adult athletic club: six track and field athletes (high aerobic capacity), five triathlon athletes (high aerobic and anaerobic capacity), and two volleyball players (high anaerobic capacity). We confirmed that they were free of any health problems and did not habitually smoke or take medication. We asked them to refrain from strenuous exercise the day before and on the day of each testing session. Moreover, we instructed the participants not to consume food or caloric beverages after 21:00 on the day before each testing session. All participants provided written informed consent before participation and received monetary compensation after completing all testing sessions. This research was performed in accordance with the Declaration of Helsinki and relevant regulations. All experimental procedures were approved by the ethics committees of Showa Women’s University (17 − 03, 19–53) and the School of Pharmacy (16 − 007, 16-007-2) and College of Sports Sciences (2019-013) at Nihon University.

### Experimental procedure

All participants visited our laboratory three times (one preliminary session and two main experimental sessions) after overnight fasting. These sessions were separated by at least 3 days and conducted at approximately the same time (09:00) to minimize the effects of circadian rhythm. During the first laboratory visit, each participant’s ${\rm{\dot V}}{{\rm{O}}_2}$max was measured. During the second and third laboratory visits, the participants ingested a beverage containing ISO or SUC in a counterbalanced order, 60 min before exercise and performed 90 min of treadmill running without energy supplementation. This setup was used to separate the effects of a single dose of ISO (or SUC) from those of supplementation during exercise and to minimize the gastrointestinal discomfort caused by supplementation during exercise. The 30-s maximum power was measured by the Wingate anaerobic test immediately after completion of the 90-min endurance exercise.

**Test beverages.** The test beverage used in this study was 500 mL of water containing either 75 g ISO or 75 g SUC. ISO and SUC were provided by Mitsui DM Sugar Co. Ltd. We outsourced adjustments such as ensuring the two beverages contained the same amount of sugar and looked and smelled similar to a specialized company (Kantou Shokuken Co., Ltd.). Its components were water (84.82%), ISO or SUC (15%), anhydrous citric acid (0.1%), and trisodium citrate (crystal; 0.08%). None of the participants could differentiate between the test beverages in terms of appearance or smell.

**Preliminary session.** At the preliminary visit, participants filled out a general health questionnaire and the body weight was measured (InBody470, InBody Japan). Body mass index (BMI) was calculated using the measured body weight (kg) and self-reported height (m). After confirming that there were no physical problems or medication use on that day, participants performed a ${\rm{\dot V}}{{\rm{O}}_2}$max test on a motorized treadmill. During the ${\rm{\dot V}}{{\rm{O}}_2}$max test, the heart rate (HR) was monitored (Lifescope VS, Nihon Kohden, Tokyo, Japan), and expired gases were collected continuously. Oxygen (O_2_) and carbon dioxide (CO_2_) concentrations in the expired gases were measured using a gas measurement system (Aeromonitor AE-310 S; Minato Medical Science, Tokyo, Japan). Electrodes were attached to the participants to measure the electrocardiogram and a mask was placed to collect expired gases. The participants stood at rest on the treadmill for 1 min, and subsequently walked at 8 km/h for 1 min as a warm-up. The ${\rm{\dot V}}{{\rm{O}}_2}$max test started at a treadmill speed of 10 km/h, which was increased by 1 km/h every minute until the participants achieved volitional exhaustion. In participants who were not exhausted at a treadmill speed of 20 km/h, the treadmill slope was increased by 1% every minute. We adopted this protocol because all the participants were athletes from a variety of sports who were able to run at high speed but were not accustomed to running on an inclined treadmill. In well-trained individuals, there is no significant difference in the ${\rm{\dot V}}{{\rm{O}}_2}$max measured with different load-increment protocols for treadmill speed and/or slope [29]. Volitional exhaustion (${\rm{\dot V}}{{\rm{O}}_2}$max) was considered achieved if two of the following three criteria were met: (1) ${\rm{\dot V}}{{\rm{O}}_2}$ was stable or decreased with an increase in treadmill speed, (2) heart rate exceeded 180 bpm, and (3) respiratory quotient exceeded 1.1 [30].

**Fig. 1:**
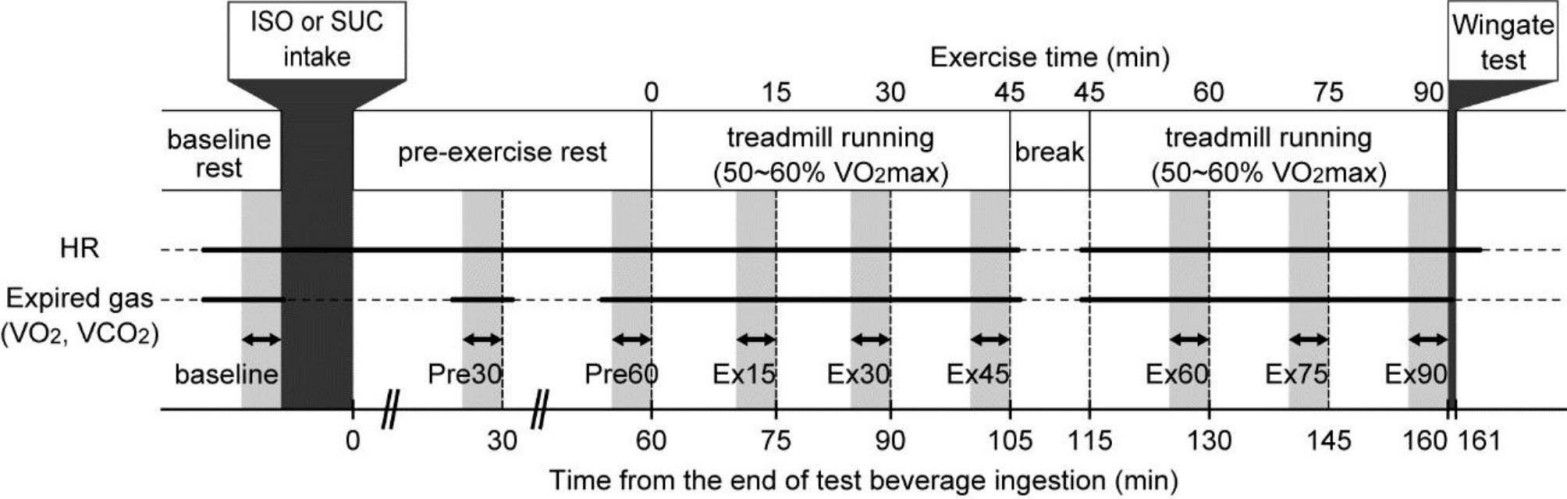
Protocol of the experimental trial. The experimental trial comprised of a baseline rest, the ingestion of a test beverage (ISO or SUC), a 60-min pre-exercise rest, a 90-min running exercise with a 10-min break in between, and a Wingate anaerobic test. Bold horizontal lines indicate recording of HR and expired gas, and dashed lines indicate no recording. Two-headed bold arrows with light gray bands indicate the time periods for steady-state HR, ${\rm{\dot V}}{{\rm{O}}_2}$, and ${\rm{\dot VC}}{{\rm{O}}_2}$ data

**Main experimental sessions**. A schematic representation of the main experimental session is shown in Fig. 1. On the experimental day, the participants visited our laboratory at 08:30 and confirmed that there were no physical problems or medication use on that day. Participants sat on a chair at rest for 10 min after preparing for measurement, and the HR and expired gases (${\rm{\dot V}}{{\rm{O}}_2}$ and ${\rm{\dot VC}}{{\rm{O}}_2}$) at rest were measured as baseline data. Subsequently, the participants removed their masks (for the measurement of expired gases) and ingested 500 mL of the test beverage. The participants were not informed which test beverage was provided. In this study, we defined the end time of test beverage intake as the start time of the pre-exercise rest period. The participants remained seated on a chair at rest for 60 min. The expired gas levels at the middle and end of the pre-exercise rest period (Pre30 and Pre60) were measured by attaching a mask. After completing the 60-min of pre-exercise rest, the participants ran on a treadmill for 90 min while wearing the mask. One of the authors (NO) gradually increased the treadmill speed during the first 1–2 min of the exercise period and adjusted the treadmill speed until the individual target ${\rm{\dot V}}{{\rm{O}}_2}$ level (60% ${\rm{\dot V}}{{\rm{O}}_2}$max of the participant) was achieved. Thereafter, the participants’ ${\rm{\dot V}}{{\rm{O}}_2}$ was continuously monitored to adjust the treadmill speed to ensure that the target ${\rm{\dot V}}{{\rm{O}}_2}$ level is maintained during the 90-min exercise period. The HR and expired gases (${\rm{\dot V}}{{\rm{O}}_2}$ and ${\rm{\dot VC}}{{\rm{O}}_2}$) were continuously recorded during exercise. Forty-five minutes after starting the treadmill running, a 10-min break was provided. This break time was provided because 90 min of mask-on exercise without hydration or a restroom break is too long. During the break, the participants removed their masks and were allowed to drink plain water ad libitum. Participants consumed 163 ± 128 mL of water in the ISO condition and 186 ± 119 mL of water in the SUC condition during this break. They were also instructed to use the restroom if necessary (three participants in the ISO condition and five participants in the SUC condition). After a 10-min break, participants ran again on a treadmill for the remaining 45 min. The treadmill speed was adjusted in the same manner as in the first half to ensure that the target ${\rm{\dot V}}{{\rm{O}}_2}$ level was maintained. Immediately after the 90-min treadmill running exercise, the participants removed their masks and rode on a bicycle ergometer (Power Max; Konami Corp., Tokyo, Japan) next to the treadmill and performed a 30-s Wingate anaerobic test. In this test, participants were instructed to pedal at maximal speed for 30 s against individual loads equal to 7.5% of their body weight [24]. Verbal encouragement from one of the authors (NO) was provided during the test to ensure that the participants performed at their maximal cycling capacity.

### Data processing

During the baseline and pre-exercise rest periods (baseline, Pre30, and Pre60; Fig. 1), HR [bpm] and expired gases (${\rm{\dot V}}{{\rm{O}}_2}$ and ${\rm{\dot VC}}{{\rm{O}}_2}$ [L/min]) were measured for 10 min, and the average values during the last 5 min were used for data analysis. During the 90-min treadmill running exercise, HR and expired gases (${\rm{\dot V}}{{\rm{O}}_2}$ and ${\rm{\dot VC}}{{\rm{O}}_2}$) were continuously measured, and we used averaged data during the 5 min just before the following six time points: 15, 30, 45, 60, 75, and 90 min after starting the treadmill running (Ex15, Ex30, Ex45, Ex60, Ex75, and Ex90; Fig. 1). ${\rm{\dot V}}{{\rm{O}}_2}$ and ${\rm{\dot VC}}{{\rm{O}}_2}$ were transformed into energy expenditure (EE [kcal/min]) data using the Weir formula [31] and calculated in kcal per kilogram of body weight [kcal/kg/min]. During rest [32] and moderate- to high-intensity exercise [33], stoichiometric equations were used to calculate the CHO and fat oxidation rates [g/kg/min]. From these data, we calculated the EE for each energy substrate [kcal/kg/min] and estimated the percentage of CHO-derived EE of the total EE. We also estimated the percentage of fat-derived EE [%] as the residual EE after determining the CHO-derived EE [%].

To examine the details of the decrease in CHO-derived EE over time after CHO intake, the slope and y-intercept of the regression line were calculated for each participant and condition using the CHO-derived EE values at eight time periods other than the baseline (before intake). The slope indicates the rate of decrease in CHO-derived EE per hour [%/h]. The y-intercept indicates the CHO-derived EE [%] at the time of exercise onset (time = 0).

During the Wingate anaerobic test, a sharp rise in the first 10 s and an exponential decline in the remaining 20 s were observed in the participants’ power output during pedaling [34, 35]. From the 30-s power output data, the peak power, mean power, time-to-peak power, and fatigue index were measured according to accepted procedures [24, 36]. The peak power was the highest mechanical power observed at a mean of 5-s epochs. The mean power corresponds to the mean power output maintained during the 30-s test. The time-to-peak power (s) corresponds to the time from the start of cycling to the peak revolutions per minute (RPM). Peak and mean power were recorded in watts [W] and calculated in watts per kilogram of body weight [W/kg]. The fatigue index [%] was defined as the percentage drop in the power output from the highest to the lowest 5-s segment.

### Statistical analysis

As a prerequisite for comparing the physiological and metabolic data during the 90-min treadmill running exercise and the performance of the subsequent Wingate anaerobic test between the two test beverage conditions, it was necessary to confirm whether the experiment was conducted as planned. Therefore, we first conducted paired t-tests on the exercise parameters (average speed [km/h], total distance [km], and average % ${\rm{\dot V}}{{\rm{O}}_2}$max during the 90-min treadmill running exercise).

Subsequently, we applied repeated measures two-way analysis of variances (ANOVAs) to the physiological and metabolic data. We expected that the physiological and metabolic data (HR, EE, CHO oxidation rate, and fat oxidation rate) would change substantially between the rest and exercise periods. Applying one-way ANOVA to all the data during the rest and exercise periods obscures the effects of time and test beverage due to the extreme changes between the two conditions. To clarify the effects of time and test beverage consumption on these data at rest and during exercise, we applied separate ANOVAs for the data during rest and exercise. For the pre-exercise rest period data, repeated measures 2 × 3 ANOVAs were conducted with the two test beverages (ISO or SUC) and three time periods (baseline, Pre30, and Pre60) as within-participant factors. For the 90-min treadmill running exercise data, repeated measures 2 × 6 ANOVAs were conducted with the two test beverages and six time periods (Ex15, Ex30, Ex45, Ex60, EX75, and Ex90) as within-participant factors. However, because variables of CHO-derived EE were normalized to a percentage of the total energy consumption at each time point, they would not be clearly different between rest and exercise. As a result, repeated measures 2 × 9 ANOVAs were conducted for CHO-derived EE, with the two test beverages and nine time periods as within-participant factors. For all repeated measures ANOVA, we used Mauchly’s test to evaluate the sphericity assumption. When necessary, the Greenhouse-Geisser procedure was used to correct the degrees of freedom. The slope and y-intercept values of the regression lines calculated from the time course of the CHO-derived EE values under the ISO and SUC conditions were compared using paired t-tests.

Finally, we conducted paired t-tests on the performance data of the Wingate anaerobic test (peak power, mean power, time-to-peak power, and fatigue index) under ISO and SUC conditions. Statistical analysis was performed using SPSS 25.0 for Windows (IBM SPSS, Chicago, IL, USA). For all tests, the level of significance was set at *p* < 0.05. Data are presented as mean ± SD.

## Results

### Exercise parameters during the 90-min treadmill running

In Table 1, exercise parameters during the 90-min treadmill running after ISO and SUC intake are shown. There were no significant differences in these exercise parameters between the ISO and SUC conditions (average speed: *t*_[12]_ = 1.41, *p* = 0.18, *d* = 0.41; total distance: *t*_[12]_ = 1.45, *p* = 0.17, *d* = 0.41; average % ${\rm{\dot V}}{{\rm{O}}_2}$max: *t*_[12]_ = 0.77, *p* = 0.46, *d* = 0.21). During the 90-min treadmill running exercise and subsequent Wingate test, none of the participants complained of gastrointestinal discomfort.

**Table 1:** Exercise parameters during the 90-min treadmill running

	ISO	SUC
Average speed (km/h)	12.2 ± 2.0	12.1 ± 1.9
Total distance (km)	18.4 ± 3.1	18.2 ± 3.0
Average % ${\rm{\dot V}}{{\rm{O}}_2}$max	62.1 ± 6.3	61.1 ± 5.1

Data presented as mean ± SD

### Physiological and metabolic parameters during pre-exercise rest

**Fig. 2:**
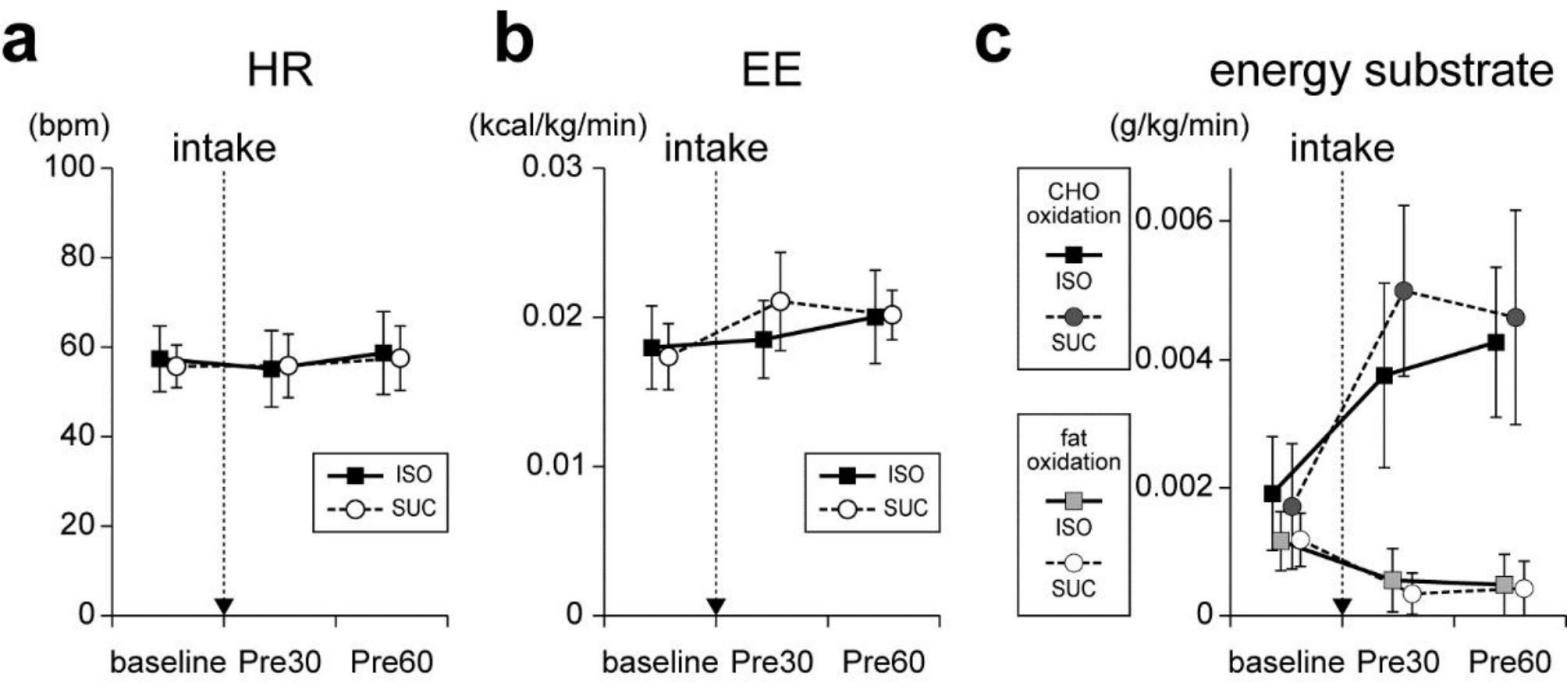
Physiological and metabolic parameters during pre-exercise rest. (**a**) HR [bpm], (**b**) EE [kcal/kg/min], and (**c**) CHO and fat oxidation rates [g/kg/min] in ISO and SUC conditions. The downward arrow indicates CHO intake. All values are presented as means ± SD (n = 13)

The HR during three pre-exercise rest periods (baseline, Pre30, and Pre60) in the ISO and SUC conditions are shown in Fig. 2a. A repeated measures two-way ANOVA on the HR during pre-exercise rest revealed no significant main effect of test beverage (*F*_[1, 12]_ = 0.38, *p* = 0.55, *η*^*2*^_*p*_ = 0.03) or time period (*F*_[2, 24]_ = 3.19, *p* = 0.06, η^2^_p_ = 0.21), and no significant interaction of test beverage × time period (*F*_[2, 24]_ = 1.78, *p* = 0.19, *η*^*2*^_*p*_ = 0.13). This result indicates that the HR did not change similarly during the pre-exercise rest period after ISO and SUC intake.

The EE during the three pre-exercise rest periods in the ISO and SUC conditions are shown in Fig. 2b. A repeated measures two-way ANOVA on the EE during pre-exercise rest revealed no significant main effect of test beverage (*F*_[1, 12]_ = 0.99, *p* = 0.34, *η*^*2*^_*p*_ = 0.08). However, there was a significant main effect of time period (*F*_[2, 24]_ = 10.68, *p* < 0.01, *η*^*2*^_*p*_ = 0.47) and a significant interaction of test beverage × time period (*F*_[2, 24]_ = 4.85, *p* < 0.05, *η*^*2*^_*p*_ = 0.29). This result indicates that EE changed differently during the pre-exercise rest period after ISO and SUC intake.

The CHO and fat oxidation rates during the three pre-exercise rest periods under the ISO and SUC conditions are shown in Fig. 2c. A repeated measures two-way ANOVA on the CHO oxidation rate during pre-exercise rest revealed that a main effect of test beverage was not significant (*F*_[1, 12]_ = 2.08, *p* = 0.18, *η*^*2*^_*p*_ = 0.15). However, a main effect of time period (*F*_[1.98, 23.72]_ = 56.55, *p* < 0.01, *η*^*2*^_*p*_ = 0.83) and an interaction of test beverage × time period (*F*_[1.46, 17.49]_ = 7.47, *p* < 0.01, *η*^*2*^_*p*_ = 0.38) was significant. A repeated measures two-way ANOVA on the fat oxidation rate during pre-exercise rest revealed that a main effect of test beverage (*F*_[1, 12]_ = 0.20, *p* = 0.66, *η*^*2*^_*p*_ = 0.02) and an interaction of test beverage × time period (*F*_[2, 24]_ = 1.96, *p* = 0.16, *η*^*2*^_*p*_ = 0.14) were not significant. However, a main effect of time period (*F*_[2, 24]_ = 62.99, *p* < 0.01, *η*^*2*^_*p*_ = 0.84) was significant. These results indicate that the CHO oxidation rate changed differently, and the fat oxidation rate changed similarly, during the pre-exercise rest period after ISO and SUC intake.

### Physiological and metabolic parameters during endurance exercise

**Fig. 3:**
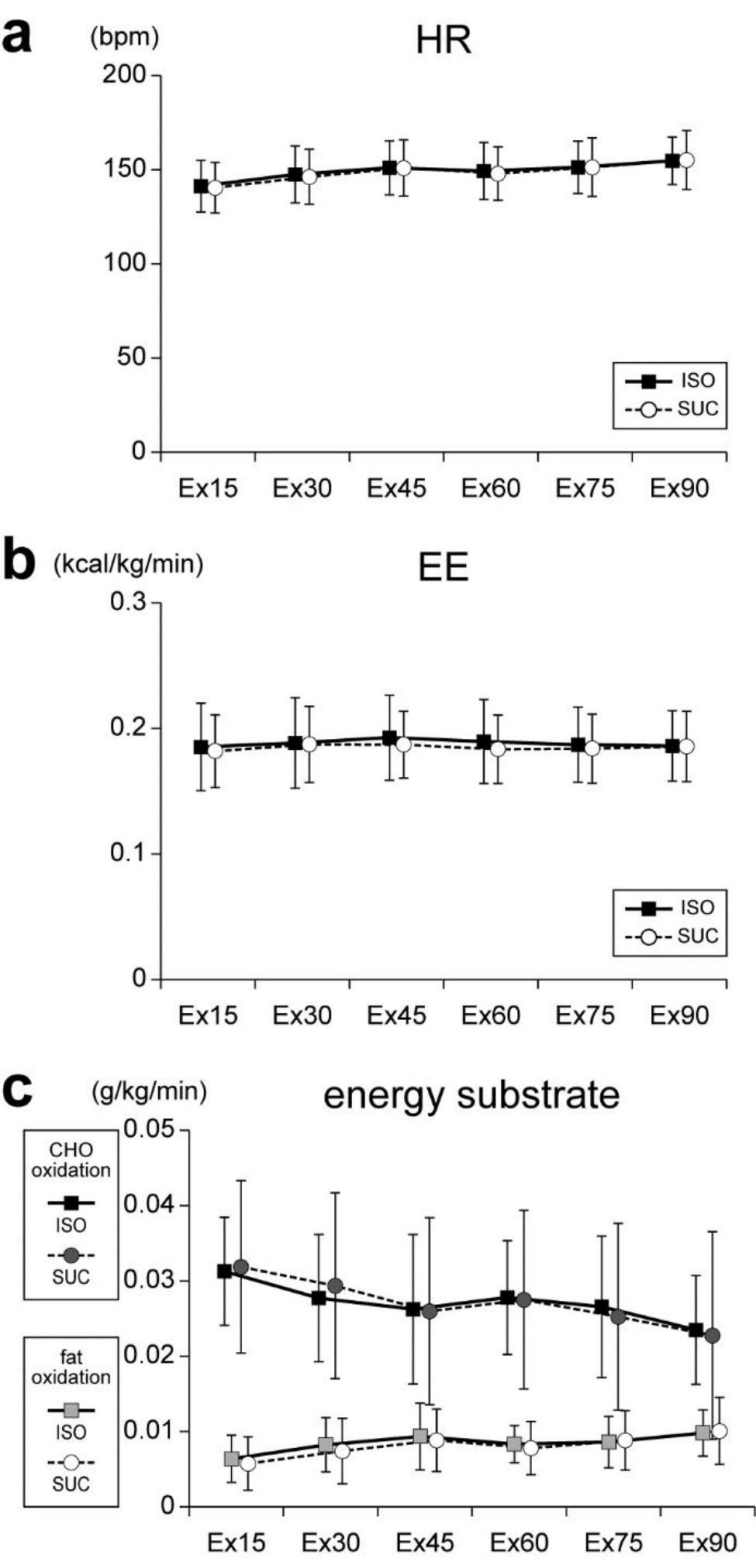
Physiological and metabolic parameters during exercise. **(a)** HR [bpm], **(b)** EE [kcal/kg/min], and **(c)** CHO and fat oxidation rates [g/kg/min] in ISO and SUC conditions. All values are presented as means ± SD (n = 13)

The HR during six treadmill running exercise periods (Ex15, Ex30, Ex45, Ex60, Ex75, and Ex90) in the ISO and SUC conditions are shown in Fig. 3a. A repeated measures two-way ANOVA on the HR during treadmill running revealed that a main effect of test beverage (*F*_[1, 12]_ = 0.20, *p* = 0.66, *η*^*2*^_*p*_ = 0.02) and an interaction of test beverage × time period (*F*_[5, 60]_ = 0.39, *p* = 0.85, *η*^*2*^_*p*_ = 0.03) were not significant. However, a main effect of time period (*F*_[2.05, 24.64]_ = 56.49, *p* < 0.01, *η*^*2*^_*p*_ = 0.83) was significant. This result indicates that during endurance exercise period, the HR gradually increased similarly after ISO and SUC intake.

The EE during six treadmill running exercise periods in the ISO and SUC conditions are shown in Fig. 3b. A repeated measures two-way ANOVA on the EE during treadmill running revealed no significant main effect of test beverage (*F*_[1, 12]_ = 1.06, *p* = 0.33, *η*^*2*^_*p*_ = 0.08) or time period (*F*_[1.78, 21.34]_ = 3.48, *p* = 0.05, η^2^_p_ = 0.23), and no significant interaction of test beverage × time period (*F*_[1.87, 22.45]_ = 1.45, *p* = 0.26, *η*^*2*^_*p*_ = 0.11). This result indicates that despite a gradual increase in HR, EE did not change during the endurance exercise period after ISO and SUC intake.

The CHO and fat oxidation rates during six treadmill running exercise periods in the ISO and SUC conditions are shown in Fig. 3c. A repeated measures two-way ANOVA on the CHO oxidation rate during treadmill running revealed that a main effect of test beverage (*F*_[1, 12]_ = 0.00, *p* = 0.98, *η*^*2*^_*p*_ = 0.00) and an interaction of test beverage × time period (*F*_[2.63, 31.55]_ = 1.22, *p* = 0.32, *η*^*2*^_*p*_ = 0.09) were not significant. However, a main effect of time period (*F*_[5, 60]_ = 31.80, *p* < 0.01, *η*^*2*^_*p*_ = 0.72) was significant. A repeated measures two-way ANOVA on the fat oxidation rate during treadmill running revealed that a main effect of test beverage (*F*_[1, 12]_ = 0.05, *p* = 0.82, *η*^*2*^_*p*_ = 0.00) and an interaction of test beverage × time period (*F*_[5, 60]_ = 1.29, *p* = 0.28, *η*^*2*^_*p*_ = 0.10) were not significant, but a main effect of time period (*F*_[5, 60]_ = 29.52, *p* < 0.01, *η*^*2*^_*p*_ = 0.71) was significant. These results indicate that during the endurance exercise, the CHO oxidation rate gradually decreased and the fat oxidation rate gradually increased after ISO and SUC intake.

### Percentages of CHO-derived EE

**Fig. 4:**
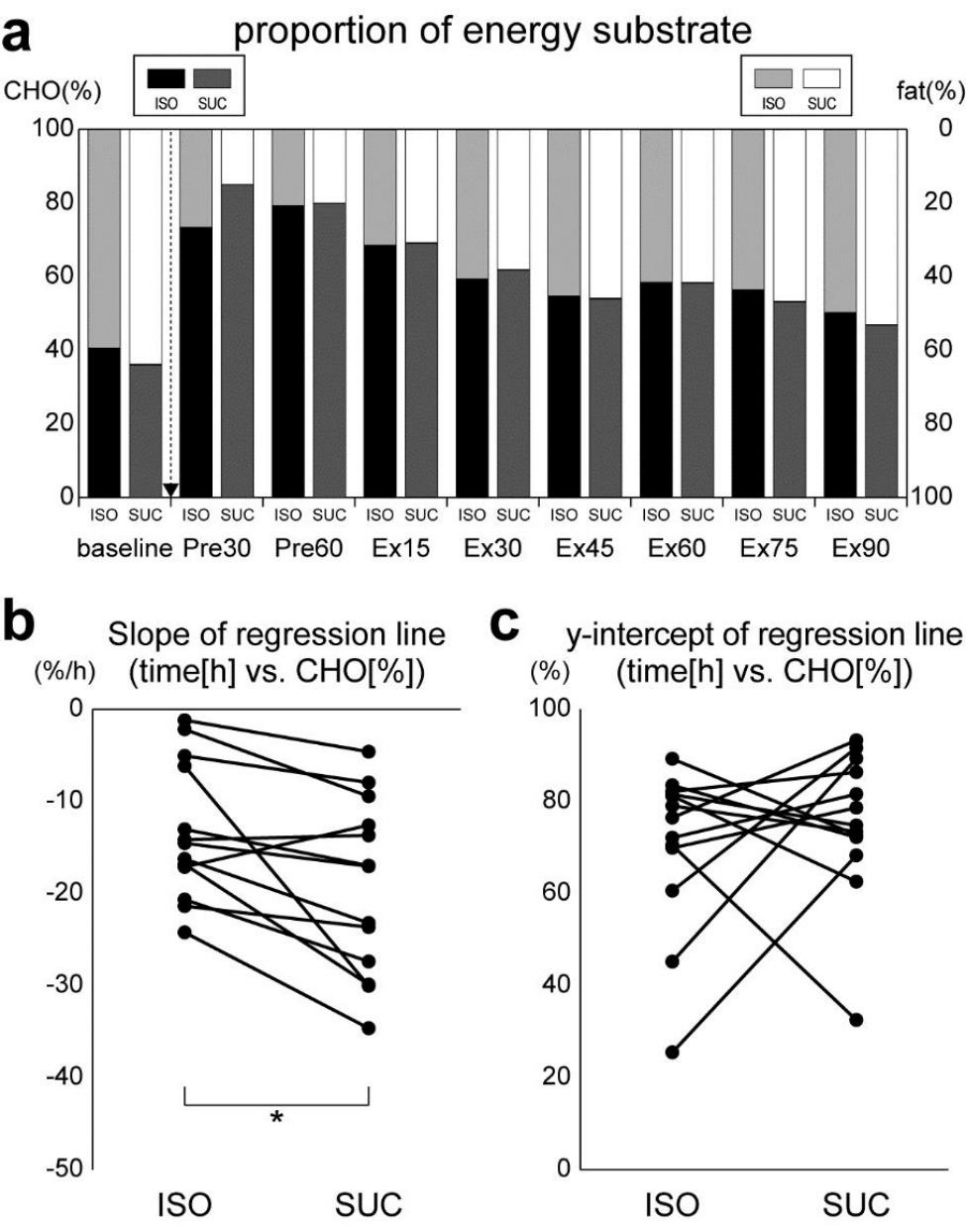
Changes in the percentages of CHO-derived EE throughout pre-exercise rest and exercise. (**a**) CHO- and fat-derived EE [%] in ISO and SUC conditions. The downward arrow indicates CHO intake. **(b)** Slopes [%/h] and **(c)** Y-intercepts [%] of the regression lines in ISO and SUC conditions for all participants (n = 13). Slopes and y-intercept of the regression lines are calculated from the CHO-derived EE at eight time periods other than baseline. * *p* < 0.05

Percentages of CHO-derived EE (and fat-derived EE) during the nine time periods throughout pre-exercise rest and exercise in the ISO and SUC conditions are shown in Fig. 4a. The CHO-derived EE increased immediately after CHO intake (Pre30) and gradually decreased throughout subsequent rest and exercise periods under both ISO and SUC conditions. A repeated measures two-way ANOVA on the proportion of energy substrate throughout pre-exercise rest and exercise revealed that a main effect of test beverage was not significant (*F*_[1, 12]_ = 0.01, *p* = 0.94, *η*^*2*^_*p*_ = 0.00). However, a main effect of time period was significant (*F*_[2.40, 28.79]_ = 32.34, *p* < 0.01, *η*^*2*^_*p*_ = 0.73) and an interaction of test beverage × time period was marginally significant (*F*_[3.40, 40.74]_ = 2.45, *p* = 0.07, *η*^*2*^_*p*_ = 0.17). This result demonstrates that CHO-derived EE increased just after the test beverage intake (Pre30) and gradually decreased during subsequent rest and exercise periods. However, this modulation pattern might differ between the ISO and SUC conditions.

To further examine the difference in the time course of CHO-derived EE between the ISO and SUC conditions, we calculated the regression line for each participant and condition using the CHO-derived EE at eight time periods other than the baseline. The slope and y-intercept of the regression lines were then compared between the ISO and SUC conditions. Paired *t*-tests revealed that the slope in the SUC condition was significantly (negatively) larger than that in the ISO condition (*t*_[12]_ = 3.10, *p* < 0.01, *d* = 0.89; Fig. 4b). However, there was no significant difference in the y-intercept between the ISO and SUC conditions (*t*_[12]_ = 0.69, *p* = 0.51, *d* = 0.19; Fig. 4c). These results indicate that differences in the time courses of CHO-derived EE throughout the rest and exercise periods after ISO or SUC intake in the main experiment were observed in the slope but not in the y-intercept.

### Performance in Wingate anaerobic test after exercise

**Fig. 5:**
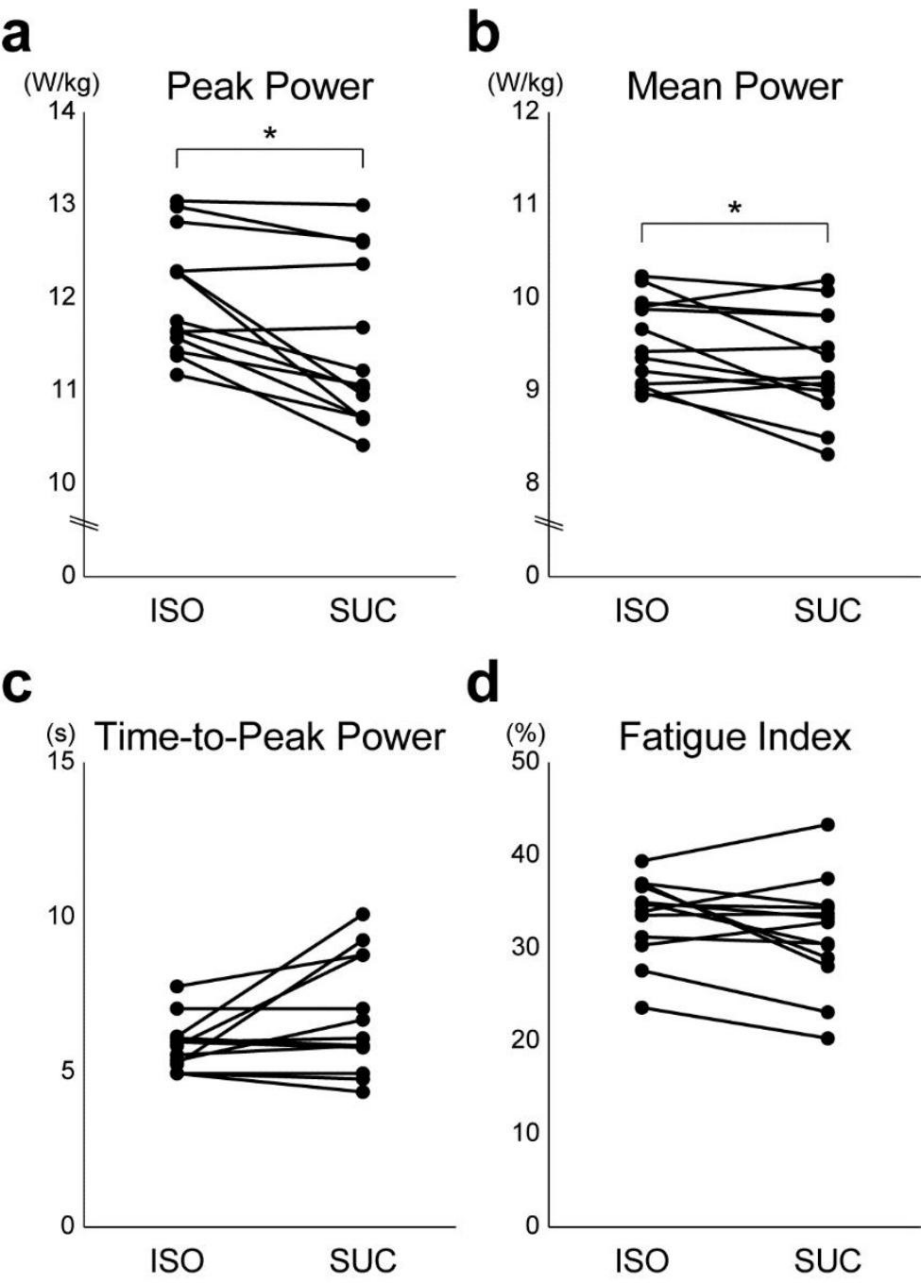
Performance in Wingate anaerobic test after exercise. (**a**) Peak power [W/kg], **(b)** mean power [W/kg], **(c)** time-to-peak power [%], and **(d)** fatigue index [%] in ISO and SUC conditions for all participants (n = 13). * *p* < 0.05

For performance in the Wingate anaerobic test, paired *t*-tests revealed that peak power (*t*_[12]_ = 3.90, *p* < 0.01, *d* = 1.19; Fig. 5a) and mean power (*t*_[12]_ = 2.42, *p* < 0.05, *d* = 0.70; Fig. 5b) values in the ISO condition were significantly larger than those in the SUC condition. However, there were no significant differences in the time-to-peak power (*t*_[12]_ = 2.11, *p* = 0.06, *d* = 0.65; Fig. 5c) and fatigue index (*t*_[12]_ = 1.67, *p* = 0.12, *d* = 0.49; Fig. 5d) between the ISO and SUC conditions. These results indicate that the maximal power output in the Wingate test immediately after endurance exercise was higher throughout for 30 s in the ISO condition than in the SUC condition.

## Discussion

The purpose of this study was to clarify the time course of CHO utilization during endurance exercise after ISO and SUC intake, and maximal anaerobic power output at the end of exercise. Consequently, the estimated time courses of the percentages of CHO-derived EE suggest that, under the ISO condition in comparison with the SUC condition, the rapid increase immediately after intake was suppressed, and the decline rate during subsequent endurance exercise remained small. In addition, the maximal power output in the Wingate anaerobic test immediately after endurance exercise was higher under the ISO condition than under the SUC condition. To the best of our knowledge, this is the first study to identify the effects of ISO or SUC intake on maximal anaerobic power output at the end of endurance exercise.

In this study, we carefully ensured that all participants exercised at the planned constant intensity level (50 − 60% of ${\rm{\dot V}}{{\rm{O}}_2}$max) in the ISO and SUC conditions. Despite the strict setting of equivalent-intensity exercise, the HR during endurance exercise increased gradually under both intake conditions. This is most likely due to a cardiovascular drift, which is defined as an increase in HR and a decrease in stroke volume over time during exercise in heat-stressed environments [37, 38]. However, as this drift generally does not significantly alter cardiac output, the EE during the endurance exercise in this study did not demonstrate a significant change over time. In contrast, during the 60 min of pre-exercise rest after ISO or SUC intake, EE increased despite remaining in the resting state, and the changing pattern of EE was different in the ISO and SUC conditions. This is probably due to diet-induced thermogenesis (or the thermic effect of food), which is defined as an increase in energy expenditure associated with a specific dynamic action of food (and/or drink) intake, including digestion and absorption [39–41]. Rapid digestion and absorption of SUC results in a rapid increase in EE, while slow digestion and absorption of ISO suppress the increase in EE [42]. As a result, the interaction between the test beverage and time period appeared in the EE during pre-exercise rest after ISO and SUC intake.

The EE during endurance exercise after ISO or SUC intake was maintained at a nearly constant level, whereas the energy substrates (CHO and fat) changed significantly over time. The slow digestion and absorption of ISO result in lower glycemic and insulinemic responses, leading to a lower CHO oxidation rate and a larger fat oxidation rate after ISO intake [17]. König et al. [20, 43] reported that ISO intake suppresses CHO oxidation rate and facilitates fat oxidation rate during the subsequent rest and exercise. In contrast to previous studies, the present study showed that the CHO oxidation rate gradually decreased (and the fat oxidation rate gradually increased) under both ISO and SUC conditions. Moreover, ANOVA for the percentages of CHO-derived EE during the entire experimental period showed a near-significant interaction between the test beverage and time period. The difference in CHO utilization over time was reflected more clearly by the fact that the slope of the linear regression line in the time course of CHO-derived EE was significantly (negatively) larger in the SUC condition than in the ISO condition. This is similar to the findings of Wong et al. [44] where a high-GI meal immediately produced a sharper increase in blood glucose levels than a low-GI meal intake did, which became similar after two-hours of rest, and decreased after approximately 100 min of running.

Furthermore, we compared the performance of a 30-s Wingate anaerobic test immediately after completing a prolonged endurance exercise between the ISO and SUC conditions. The results showed that both the peak power and mean power were greater under the ISO condition than under the SUC condition. However, there was no difference in either the time-to-peak power or fatigue index. The difference in the rate of decrease in the percentage of CHO-derived EE during endurance exercise after ISO or SUC intake indicate that a high percentage of CHO-derived EE may have been maintained at the end of the endurance exercise in the ISO condition. Therefore, the difference in mean power could be influenced by the contribution of anaerobic glycolysis, in which only CHO is involved. Thus, the results of this study appear to be in line with our hypothesis.

The difference in peak power and mean power and no difference in either time-to-peak power or fatigue index between the ISO and SUC conditions cannot be explained by the difference in CHO-derived EE percentage alone. Based on the difference in the CHO-derived EE percentage alone, we can assume that the difference in the power output during the first few seconds of the 30-s period when the ATP-PCr system’s contribution dominates is minimal, and this difference becomes more apparent after the mid-period when the contribution of anaerobic glycolysis increases. Thus, there may be a difference in the fatigue index, which is the rate of decrease in power output within 30 s. However, the power output, including the peak power, decreased throughout the 30-s Wingate test in the present study. This suggests that factors other than the amount of available CHO may affect the difference in anaerobic power output after prolonged exercise.

Fritzsche et al. [45] found that the ingestion of CHO-containing water before and during prolonged exercise promoted CHO oxidation by maintaining high blood glucose levels, and reduced the decline in short-term maximal power output during cycling. Low blood glucose level-induced decrease in CHO oxidation during prolonged exercise reportedly increases blood lactate and perceived exertion levels [5]. Considering these findings, the difference in the decline in CHO oxidation between the ISO and SUC conditions in this study suggests that differences in blood lactate concentrations and perceived output levels may have occurred at the end of the prolonged exercise. This implies that in addition to CHO depletion, the neuromuscular and central nervous systems may be differently affected, resulting in differences in peak power. As these detailed mechanisms cannot be clarified from our study results, future research focusing on these mechanisms is required.

This study had several methodological limitations. First, the sample size was small. For paired t-tests in our experimental setting (effect size dz [large] = 0.8, α = 0.05, power = 0.8), the sample size was a priori estimated to be 15 using G*power [46]. In contrast, the statistical power values of the paired t-test computed from our data, which were judged to be significantly different, were 0.60 − 0.95. Owing to the small sample size and low statistical power, our study results should be cautiously interpreted. Additionally, our sample was restricted to young male athletes. This is because performing high-intensity exercises for 90 min in untrained athletes is difficult. Further research should be conducted with a more diverse sample, especially in female athletes, who were not included in this study. Second, we investigated metabolism at rest and during exercise after ISO or SUC intake by measuring the expired gas. The responses in blood glucose and insulin concentrations after ISO or SUC intake were discussed based on changes in CHO and fat oxidation rates calculated from the expired gas with reference to previous studies [7, 16, 17, 19, 20, 22, 43, 47]. It is desirable to collect and measure participants’ blood samples simultaneously, which would further strengthen the results of this study.

This study has several implications for the future. To clarify the effects of a single dose of ISO or SUC in this study, participants took them only 60-min before exercise onset and did not receive supplementation afterwards. The participants fasted overnight, and the amount of CHO oxidized during the entire experimental period after intake was theoretically greater than the amount of CHO ingested in this experiment. Therefore, the effects of single doses of ISO or SUC were investigated. However, in an actual competitive scenario, athletes may be able to supplement CHO frequently during exercise [19, 22]. Febbraio et al. [47] showed that pre-exercise CHO intake increased the CHO oxidation rate and that post-exercise CHO oxidation rate levels could be maintained at high levels with regular CHO supplementation but declined drastically without CHO supplementation. Given the potential risk of gastrointestinal discomfort [22] and the desire for an immediate effect in maintaining CHO levels during the final stage of various sports games, fast-digesting CHOs may be more appropriate than ISO for supplementation during prolonged exercise. Thus, it is necessary to further investigate the optimal intake method for athletes based on various combinations of ISO intake amount, intake timing (before or during exercise), and intake method (single or combined).

In summary, compared with SUC intake, after ISO intake, the percentage of CHO-derived EE during prolonged endurance exercise did not decline abruptly but remained relatively high until the final exercise phase. Additionally, anaerobic power output at the end of the exercise, largely contributed by anaerobic glycolysis, was greater after ISO intake than after SUC intake. Although the results of this study suggest a suitable CHO intake to improve performance in the final phase of various sports competitions (e.g., just before the finish line in long-distance running or during additional time in the second half of a soccer game), further studies are needed to establish its validity and reproducibility.

## Data Availability

Data are available from the corresponding author upon reasonable request.

## Abbreviations

ANOVA: Analysis of variance
ATP-PCr: Adenosine triphosphate-phosphocreatine
CHO: Carbohydrate
EE: Energy expenditure
GI: Glycemic index
HR: Heart rate
ISO: Isomaltulose
RPM: revolutions per minute
SD: Standard deviation
SUC: Sucrose
${\rm{\dot VC}}{{\rm{O}}_2}$: Carbon dioxide production
$${\rm{\dot V}}{{\rm{O}}_2}$$: Oxygen consumption
${\rm{\dot V}}{{\rm{O}}_2}$max: Maximal oxygen consumption
